# Supplementary material for: Modeling Spatial Correlation of Transcripts with Application to Developing Pancreas
Source: Sci Rep. 2019 Apr 3;9:5592. doi: 10.1038/s41598-019-41951-2 (PMC6447534; doi:10.1038/s41598-019-41951-2)
Supplement: Supplementary file 1 — Supplementary Information [file 41598_2019_41951_MOESM1_ESM.pdf]

Modeling Spatial Correlation of Transcripts With Application to Developing Pancreas

Ruishan Liu, Marco Mignardi, Robert Jones, Martin Enge, Seung K Kim, Stephen R Quake, James Zou

Supplementary Information

Supplementary Notes

1 In Situ RNA Sequencing Data of Pancreas

To demonstrate the use of our computational analysis tool, we study in situ RNA sequencing of human fetal pancreatic tissue. The study was performed on tissues from de-identified donors with informed consent, and was approved by the ethics committee of the Stanford University Institutional Review Board (IRB). All methods were performed in accordance with the relevant guidelines and regulations. The data are collected at three developmental ages — 80, 87 and 117 days post-fertilization.

Experimental procedure. Anonymized fetal pancreas tissues were OCT-embedded and immediately frozen in liquid nitrogen upon arrival and stored at -80°C. Before tissue staining, blocks were cut in 10 μm-thick sections which were collected on superfrost plus slides and stored at 80°C.

All the solution used for tissue staining were prepared using DEPC-treated or RNase-free reagents. Slides were let air dry before fixation in 4% (w/v) paraformaldehyde in PBS for 45 min at room temperature. After fixation sections were washed in PBS for 5 min at room temperature and permeabilized with 0.01% (w/v) pepsin in 0.1 M HCl for 5 min at 37°C followed by wash in PBS (5 min room temperature) and a wash in EtOH 70, 85 and 100% for one min each at room temperature.

Slides were let air dry before mounting a silicon hybridization chamber (Secure-seal, SIGMA) of different size depending on the size of the tissue section. Tissues were washed once with PBST (1x PBS, 0.05% tween-20) at room temperature before incubation in 1x RT buffer (ThermoFisher) until reverse transcription.

In situ reverse transcription was carried out adding all the cDNA primers together (Supplementary Table 7) at a final concentration of 0.3 μM each, 1x RT buffer (ThermoFisher), 0.2 μg/μl BSA (NEB), 0.5 mM dNTPs (ThermoFisher), 1 U/μl RiboLock RNase Inhibitor (ThermoFisher) and 10 U/μl Revert AID H Minus Reverse Transcriptase (ThermoFisher) and incubating the tissue at 37°C overnight. After reverse transcription, slides were washed twice in PBST and post-fixation was done with 4% (w/v) paraformaldehyde in PBS for 10 min at room temperature followed by two washes in PBST 5 min at room temperature and incubation in 1x Ampligase buffer.

Ligation was carried out adding all the 5-end phosphorylated padlock probes together (Supplementary Table 8) at a final concentration of 0.05 μM each, 1x Ampligase buffer (Epicentre), 0.2 μg/μl BSA (NEB), 50 mM KCl, 20% Formamide, 0.4 U/μl RNase H (ThermoFisher) and 0.75 U/μl Ampligase DNA ligase (Epicentre) and incubating the tissue 30 min at 37°C followed by 60 min at 45°C. After ligation slides were washed twice in PBST and incubated in 1x Phi29 DNA Polymerase buffer. Rolling circle amplification (RCA) was carried out adding 5% glycerol, 0.2 μg/μl BSA (NEB), 0.25 mM dNTPs (ThermoFisher),

1x Phi29 DNA polymerase buffer (ThermoFisher) and 1 U/μl of Phi29 DNA polymerase (ThermoFisher) and incubating the tissue at 30°C overnight. After RCA slides were washed twice in PBST and detection mix consisting of 1x hybridization buffer (2x SSC, 20% formamide), 0.1 μM of each detection probe (Supplementary table XXX) and 10 ng/μl DAPI (ThermoFisher) was added for 30 min at 37°C.

Slides were washed twice in PBST before removing the hybridization chamber and washed in EtOH 70, 85 and 100% for 5 min each at room temperature. Stained sections were air-dried and mounted with SlowFade Antifade Gold mountant (ThermoFisher) and a coverslip before imaging.

Following staining cycles were done as follow: slides were dip in 70% EtOH until the coverslip fell off. Section were then washed in 85 and 100% EtOH 5 min each and air dried. UNG treatment was done adding 1x UNG buffer (ThermoFisher), 0.2 μg/μl BSA (NEB) and 0.05 U/μl UNG (ThermoFisher) for 30 min at 37°C. Uracil cleavage was followed by was in pre-warmed 65% formamide in water for 5 min at 55°C and two washes in PBST. Second and third staining cycles and nuclear staining were performed as described above.

Image acquisition and analysis. Imaging was carried out on a Zeiss Axioplan epifluorescence microscope equipped with an Axiocam 506 mono camera (Zeiss)and filter-cubes for DAPI, FITC, Cy3 and Cy5. Each sample was imaged after every round of hybridization with a 20x/0.8 Plan-Apochromat objective (Zeiss) for a total of three rounds. Multiple fields of view were acquired with 10% overlapping and 10-15 z-stacks (about 0.5 μm step size). The resulting images were projected (maximum intensity projection, MIP) and automatically stitched using DAPI staining. MIP images were shade corrected using the automatic function in Zen Acquisition software (Zeiss) feeding a minimum intensity projection. Images were exported as 16bit grayscale images. Background subtraction was done using ImageJ software by measuring the fluorescence intensity outside the signals and removing it from the corresponding image.

Image registration was done in ImageJ as follow: images from the same round of hybridization, except for the DAPI staining, were combined (MIP). A first registration between images from different rounds of hybridization was done using the nuclei staining (DAPI images). A second registration was done using the MIPs from the other channels. Image alignment was done using MultiStackReg plugin in ImageJ using one round of hybridization (typically the first) as a reference and aligning the other rounds to the first. For every alignment a rigid transformation was applied, and a transformation matrix was saved and applied to the rest of the images.

Pre-aligned and background-corrected images were analyzed with CellProfiler 2.1.1 (rev 6c2d896) to identify nuclei,cellsand fluorescent spots (RCA). The intensity and position of RCA products were measured using the same pipeline as in Mignardi et al. (2015) [1]. The barcode decoding was obtained using the same MATLAB script as described before (Ke et al., 2013) [2] and a quality threshold was applied to the detected barcodes as described above. All the

raw images, CellProfiler pipelines and oligonucleotide sequences are accessible in Supplementary Table. 7, Supplementary Table 8 and Supplementary Table 9.

Basic statistics. The spatial distributions of transcripts from 25 different genes are retrieved. For samples collected at age 80, 87 and 117 days after fertilization, the 2D positions of SST, GLUC and INS are plotted in Supplementary Figure 1a, Supplementary Figure 1c and Figure 1a. The total number of reads after quality control is given in Supplementary Table 1.

The positions of nuclei are identified at the same time, illustrated in Supplementary Figure 1b, Supplementary Figure 1d and Figure 1b for samples at age 80, 87 and 117 days post fertilization. The total number of counts is recorded in Supplementary Table 1.

2 Algorithm for Identifying of Endocrine Islets

The endocrine islets are identified according to Algorithm 1. In this analysis, we set number of possible radius $m = 10$, maximum possible islet radius $r_{\max} = 110\ \mu m$, minimum possible islet radius $r_{\min} = 10\ \mu m$ which is around the averaged nuclei spacing.

Algorithm 1 Identification of Endocrine Islets

Input: Nuclei positions (x_i, y_i), total number of SST, GLUC and INS transcripts in ith cell n_i $(i = 1, .., N)$, maximum possible radius $r_{\max}$, minimum possible radius $r_{\min}$ and number of radius steps m.
Initiate islets region set $C = \emptyset$ and exocrine nuclei set $P_{\text{exo}} = \{i \mid 1 \leqslant i \leqslant N,\ n_i \geqslant 1\}$
for $t = 0$ to m **do**
  Islet radius $r = (1 - t/m)r_{\max} + (t/m)r_{\min}$.
  Get the number of neighboring nuclei $n_i^{(\text{neighbor})}$ within a radius r for ith nucleus, $i \in P_{\text{exo}}$.
  The temporary islets set $P_t = \{i \mid i \in P_{\text{exo}},\ n_i^{(\text{neighbor})} > (1/\#P_{\text{exo}}) \sum_{j \in P_{\text{exo}}} n_j^{(\text{neighbor})}\}$
  while P_t is not $\emptyset$ **do**
    $i^* = \arg\max_{i \in P_t} n_i^{(\text{neighbor})}$, remove i^* from P_t
    circle c^* has center (x_{i^*}, y_{i^*}) and radius r.
    if c^* do not intersect with C **then**
      Add c^* to islets set C.
      $P_{\text{exo}} = \{i \mid i \in P_{\text{exo}}, (x_i,\ y_i) \text{ not in } c^*\}$
    end if
  end while
end for

We then identified clusters of endocrine cells for all three samples. Within

each sample we identified clusters of different size and the distribution of cluster size for each sample is plotted in Supplementary Figure 2. The total number of reads inside identified endocrine regions is given in Supplementary Table 5.

3 Density Profile-based Analysis

The density profile-based analysis is carried out to capture the relation between transcripts and other morphological features of the tissue such as nuclei's position or developing pancreatic islets.

The density profiles are calculated based on kernel density estimation. Given the characteristic distance of each transcript x_i ($i = 0, ..., n$), the density ρ at distance x is estimated as

$$\rho(x) = \frac{1}{nw} \sum_{i=1}^{n} K\left(\frac{x_i - x}{w}\right). \tag{1}$$

where n is the transcripts number, K is a kernel with bandwidth w. For example, when analyzing the relation between gene expression and endocrine regions, the characteristic distance x could be the distance between the transcript and the boundary of its closest endocrine islet. For a good approximation at short distance, we use a 1D kernel with linear combination correction [3].

The difference between two density profiles $\rho_1(x)$ and $\rho_2(x)$ is characterized by symmetric Kullback-Leibler (KL) divergence

$$D(\rho_1, \rho_2) = \frac{1}{2}\left(\int_0^\infty \rho_1(x) \log \frac{\rho_1(x)}{\rho_2(x)} \mathrm{d}x + \int_0^\infty \rho_2(x) \log \frac{\rho_2(x)}{\rho_1(x)} \mathrm{d}x\right), \tag{2}$$

which averages the KL divergence from ρ_1 to ρ_2 and the divergence from ρ_2 to ρ_1.

4 Statistical Model for Spatial Correlations

4.1 Islet shape analysis.

In the experiment, the boundaries for endocrine islets can be nicely approximated by circles and the model characterizes the spatial correlations in the circular areas. We note that the statistical model does not rely on particular boundary shape and can be readily applied to non-circular cases.

To illustrate the model's performance when fitted on non-circular regions, we repeat the spatial correlation analysis on pancreatic islets approximated by squared shapes. The endocrine islets are identified as squares, following Algorithm 1 with the radius of circles replaced by the side length of squares. Supplementary Figure 5 shows that the squared boundaries do not capture the shapes of islets well and tend to include more exocrine pancreas compared to circular boundaries. We again fit the statistical model and summarize the typical results in Supplementary Table 2, in correspondence with Table 2 in the

main text — most spatial correlations among genes are fitted to be close to 1 and three pairs of genes with positive spatial correlation are identified.

4.2 Evaluation on synthetic data

In this section, we evaluate our statistical model on synthetic datasets and demonstrate its power in characterizing the spatial distribution of the expression level among different genes.

Other methods. The computational analysis on the gene-gene spatial correlations has not been addressed in literature. Recent methods such as SpatialIDE identifies the spatial variation of individual genes on their own and the gene-gene spatial correlations are not discussed [4].

We propose two other methods as comparison — a baseline model with preliminary statistics and a pairwise Strauss process model.

In the baseline, the spatial correlation between type i and type j transcripts is defined as

$$\gamma_{ij,\text{baseline}} = \frac{\rho_i(j)}{\overline{\rho}(j)}, \tag{3}$$

where $\rho_i(j)$ denotes the mean density of type j transcripts in the neighborhood of type i within radius r and $\overline{\rho}(j)$ is the mean density of type j transcripts in the whole analyzed region. The case $\gamma_{ij,\text{baseline}} > 1$ indicates that type j transcripts are more likely to appear near type i, thus a clustering effect is expected. Similarly, $0 < \gamma_{ij,\text{baseline}} < 1$ represents an inhibition effect and $\gamma_{ij,\text{baseline}} = 1$ corresponds to an independent relation.

We further compare our multitype Strauss model with a pairwise Strauss model. The spatial correlation between type i and j are modeled with Strauss process for gene i and j only, ignoring the effects of other genes.

Synthetic datasets. We generate two toy spatial transcriptome datasets I and II with known gene-gene spatial correlations, as shown in Supplementary Figure 5. Here the interaction radius is set to be 1 unit and the analyzed region is a 20 units $\times$ 20 units square. The scale unit can be related to different length measure. Taking the pancreas transcriptomic dataset as a example, 1 unit can correspond to $20\mu m$.

In dataset I, three genes A, B and C are correlated following Supplementary Figure 5a. There is a clustering effect between pairs (A, B) and (B, C), while the generation of gene B and C are independent. In the experiment, gene A is randomly sampled from a Poisson process on the squared region. Gene B and C are randomly generated in the neighborhood of gene A following Poisson process independently. The intensities for A, B and C are 0.1, 0.09 and 0.08, respectively. An example of generated spatial transcriptomic data are plotted in Supplementary Figure 5b.

In dataset II, two more genes D and E are added, as shown in Supplementary Figure 5c. There is a clustering effect between gene D and E, which are both

independent on the rest three genes. Genes A, B and C are generated following the same procedure as in dataset I. We sample gene D from a Poisson process on the whole region with intensity 0.1 and sample gene E from a Poisson process in the neighborhood of gene D with intensity 0.09, as illustrated in Supplementary Figure 5d.

Performance. Our model successfully captures the spatial correlations among genes and significantly outperforms the other two methods. The performance of fitting the baseline method, pairwise Strauss model and our proposed model on dataset I and II are given in Supplementary Table 3 and Supplementary Table 4, respectively.

Our statistical model is able to distinguish between spatial correlation and spatial co-occurrence, while the other two methods cannot. In the synthetic dataset I, gene B and C are independently generated, but they both correlate with gene A. Thus the two independent genes are also more likely to appear close to each other because of their common neighbor gene A. As shown in Supplementary Table 3, our statistical model correctly captures their independence as $\gamma_{ij} = 1.00 \pm 0.02$. However, the other two methods directly contribute the co-occurrence of B and C to their spatial correlation and yield $\gamma_{ij} > 1$ indicating a clustering effect.

When more types of transcripts are considered in synthetic dataset II, our statistical model continues to learn the correct correlations, as indicated by Supplementary Table 4. The independence between the two groups (A, B, C) and (D, E) is characterized by $\gamma_{ij} = 1$ with gene i and j from each group. The other two methods still mistakenly concludes that there is a clustering effect between the two independent genes B and C.

5 Full Results for Gene Correlation

In the experiment, the spatial correlation γ_{ij} among genes within endocrine islets are fitted with our statistical model. The full results are summarized in Supplementary Table 6.

Supplementary Figures

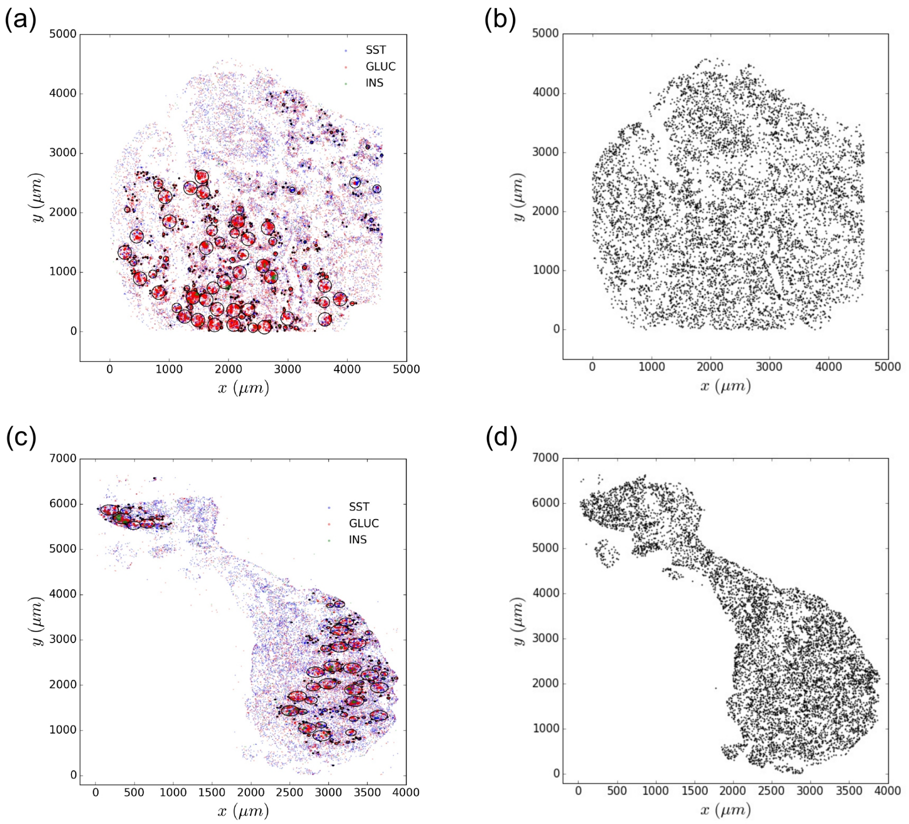

Supplementary Figure 1: **In situ sequencing.** (a)(c) Detected SST, GLUC and INS transcripts are plotted on xy coordinates for fetal pancreas sample at age (a) 80 days and (c) 87 days post fertilization. Identified pancreatic islets are identified by black circles. (b)(d) Identified and segmented nuclei are plotted on xy coordinates at age (b) 80 days and (d) 87 days post fertilization.

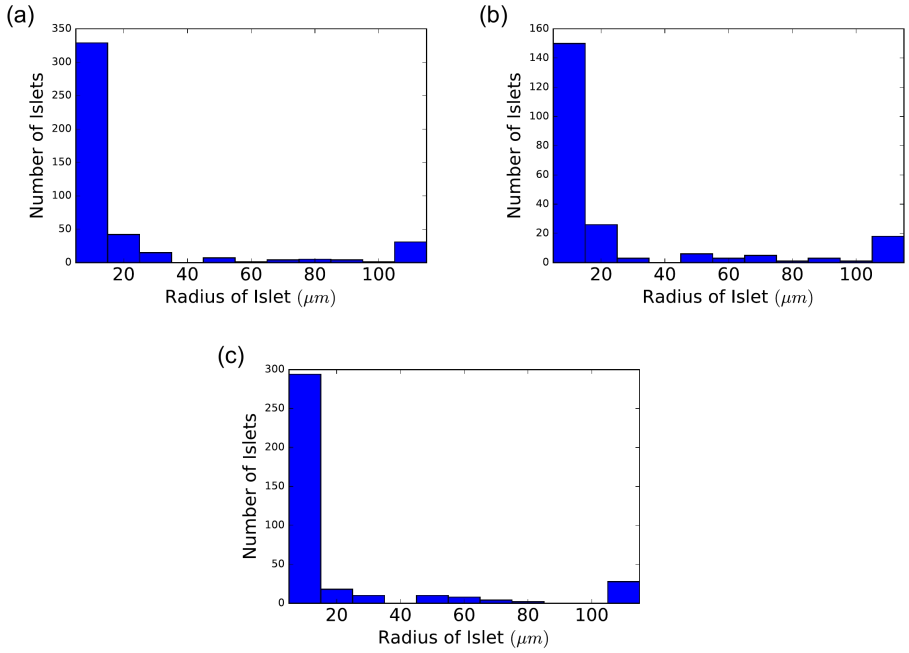

Supplementary Figure 2: **Histogram of islets radius.** The samples are collected at age (a) 80 days (b) 87 and (c) 117 days post fertilization.

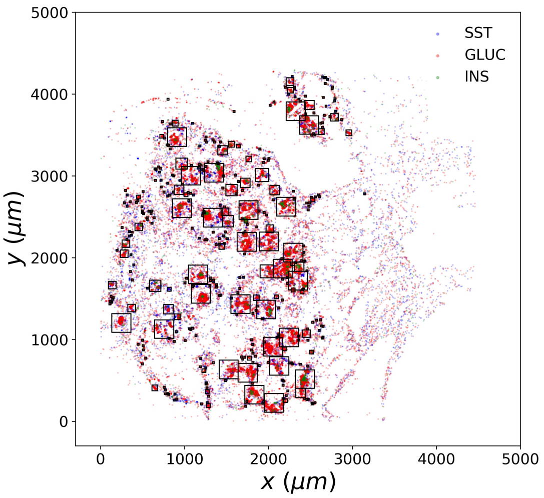

Supplementary Figure 3: **Pancreatic islets approximated by squares.** The sample is from fetal pancreas at age 117 days post fertilization. Detected SST, GLUC and INS transcripts are plotted on xy coordinates. Computationally identified pancreatic islets are identified by black squares.

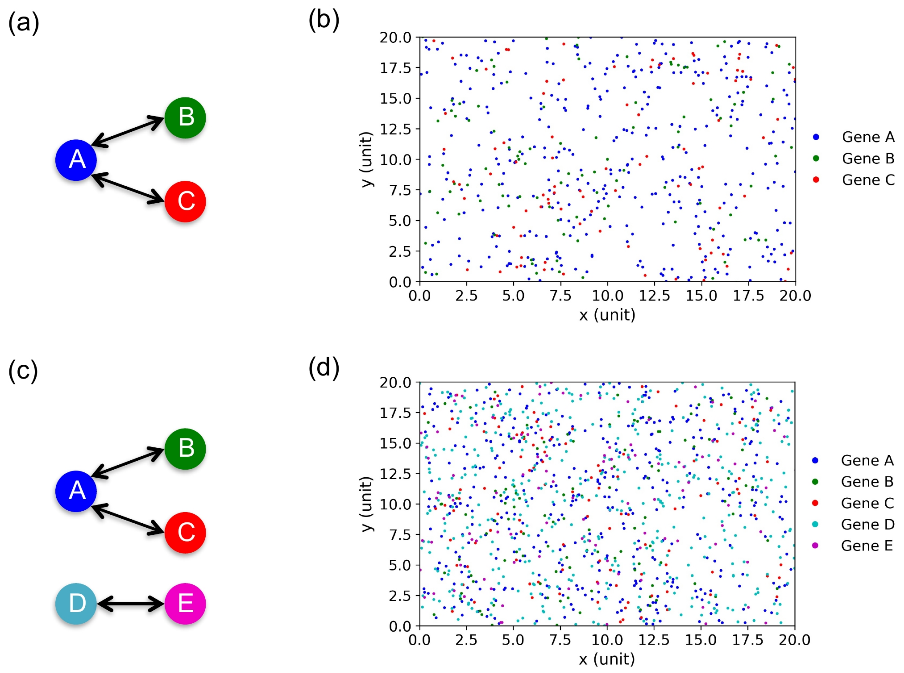

Supplementary Figure 4: **Synthetic datasets.** (a)(c) The illustration of gene correlations for (a) dataset I and (b) dataset II. The double-headed arrows connect two genes which have clustering effect with each other. If two genes are not directly connected, they are generated independently. (b)(d) The spatial transcriptomic data in (b) dataset I and (d) dataset II.

Supplementary Tables

Supplementary Table 1: Total number of different transcripts reads and nuclei at age 80, 87 and 117 days after fertilization.

	OGG1	VEGFB	TP53	SST	ARX
Day 80	1346	1151	909	28053	4952
Day 87	773	587	779	26107	12220
Day 117	875	890	601	19481	2769
	GLUC	MUC15	MUC6	MUC20	NEUROD1
Day 80	58203	12	38018	987	107
Day 87	22557	22	6182	1328	160
Day 117	50267	18	16306	725	148
	PROM1	INS	VEGFC	CDKN1A	MKI67
Day 80	3994	2642	931	231	1839
Day 87	2936	3062	841	145	7565
Day 117	2140	3412	733	99	1402
	MUTYH	PDX1	MUC16	EPCAM	MUC1
Day 80	125	452	60	5200	136
Day 87	221	1030	129	3445	208
Day 117	86	344	11	3097	75
	NEUROG3	MUC13	CDKN2A	SOD1	TOP2A
Day 80	104	103	334	2094	984
Day 87	147	31	204	1124	2067
Day 117	101	26	146	1516	515

Supplementary Table 2: When the boundaries for endocrine islets are approximated by *squares*, spatial correlation γ_{ij} ($mean \pm std$) at age 80, 87 and 117 days after fertilization.

Correlation Intensity		Day 80	Day 87	Day 117
Typical	**SST** ↔ **INS**	1.00 ± 0.02	1.00 ± 0.01	1.000 ± 0.005
	**INS** ↔ MUC6	1.00 ± 0.04	1.00 ± 0.03	0.95 ± 0.01
	**INS** ↔ ARX	1.00 ± 0.11	1.00 ± 0.03	0.89 ± 0.04
	ARX ↔ MUC6	1.00 ± 0.03	1.04 ± 0.02	1.00 ± 0.03
Strongest	EPCAM ↔ PROM1	1.22 ± 0.08	1.25 ± 0.07	1.25 ± 0.06
	MUC6 ↔ EPCAM	1.16 ± 0.03	1.14 ± 0.06	1.13 ± 0.03
	MUC6 ↔ PROM1	1.13 ± 0.03	1.13 ± 0.08	1.17 ± 0.04

Supplementary Table 3: Spatial correlation γ_{ij} ($mean \pm std$) for synthetic dataset I, fitted with three methods — the preliminary statistics baseline, the pairwise Strauss process model and our proposed statistical model. The true spatial correlation $\hat{\gamma}_{ij}$ equals to 1 when gene i and j are independent and is larger than 1 when there is a clustering effect. We randomly generate dataset I for 20 times and average the results.

Correlation Type		Baseline	Pairwise Model	Our Model
Clustering ($\hat{\gamma}_{ij} > 1$)	A ↔ B	1.37 ± 0.01	1.30 ± 0.01	1.30 ± 0.02
	A ↔ C	1.38 ± 0.01	1.33 ± 0.01	1.33 ± 0.02
Independent ($\hat{\gamma}_{ij} = 1$)	B ↔ C	$\mathbf{1.21 \pm 0.02}$	$\mathbf{1.13 \pm 0.02}$	$\mathbf{1.00 \pm 0.02}$

Supplementary Table 4: Spatial correlation γ_{ij} ($mean \pm std$) for synthetic dataset II with three methods. The true spatial correlation $\hat{\gamma}_{ij}$ equals to 1 when gene i and j are independent and is larger than 1 when there is a clustering effect. We randomly generate dataset II for 20 times and average the results.

Correlation Type		Baseline	Pairwise Model	Our Model
Clustering ($\hat{\gamma}_{ij} > 1$)	A $\leftrightarrow$ B	1.37 ± 0.01	1.30 ± 0.01	1.31 ± 0.02
	A $\leftrightarrow$ C	1.38 ± 0.01	1.33 ± 0.01	1.34 ± 0.02
	D $\leftrightarrow$ E	1.38 ± 0.02	1.32 ± 0.02	1.33 ± 0.02
Independent ($\hat{\gamma}_{ij} = 1$)	A $\leftrightarrow$ D	1.049 ± 0.006	1.008 ± 0.006	1.008 ± 0.006
	A $\leftrightarrow$ E	1.04 ± 0.02	1.00 ± 0.02	1.00 ± 0.01
	B $\leftrightarrow$ C	$\mathbf{1.21 \pm 0.02}$	$\mathbf{1.13 \pm 0.02}$	$\mathbf{1.00 \pm 0.02}$
	B $\leftrightarrow$ D	1.03 ± 0.01	1.00 ± 0.01	1.00 ± 0.01
	B $\leftrightarrow$ E	1.01 ± 0.03	1.00 ± 0.02	1.00 ± 0.02
	C $\leftrightarrow$ D	1.02 ± 0.01	1.00 ± 0.01	1.00 ± 0.01
	C $\leftrightarrow$ E	0.99 ± 0.03	0.97 ± 0.02	1.00 ± 0.03

Supplementary Table 5: Total number of reads inside endocrine regions at age 80, 87 and 117 days post fertilization.

	OGG1	VEGFB	TP53	SST	ARX
Day 80	316	183	145	14098	906
Day 87	142	121	130	13006	1937
Day 117	387	262	146	11611	697
	GLUC	MUC15	MUC6	MUC20	NEUROD1
Day 80	41274	2	6514	208	34
Day 87	14448	4	1372	280	65
Day 117	37171	4	4665	169	75
	PROM1	INS	VEGFC	CDKN1A	MKI67
Day 80	922	925	133	28	295
Day 87	812	1587	80	16	1066
Day 117	774	1904	168	23	293
	MUTYH	PDX1	MUC16	EPCAM	MUC1
Day 80	31	123	19	1252	18
Day 87	43	265	19	998	27
Day 117	23	129	5	1056	18
	NEUROG3	MUC13	CDKN2A	SOD1	TOP2A
Day 80	24	10	69	418	188
Day 87	36	7	38	208	349
Day 117	40	1	37	394	118

Supplementary Table 6: Spatial correlation γ_{ij} ($mean \pm std$) at age 80, 87 and 117 days after fertilization.

	Day 80	Day 87	Day 117
**SST** $\leftrightarrow$ **INS**	1.00 ± 0.02	1.00 ± 0.01	1.00 ± 0.01
**INS** $\leftrightarrow$ **GLUC**	1.000 ± 0.004	1.00 ± 0.02	1.011 ± 0.002
**GLUC** $\leftrightarrow$ **SST**	1.005 ± 0.001	1.003 ± 0.002	1.008 ± 0.001
EPCAM $\leftrightarrow$ PROM1	1.26 ± 0.08	1.26 ± 0.07	1.33 ± 0.09
MUC6 $\leftrightarrow$ EPCAM	1.15 ± 0.03	1.17 ± 0.08	1.12 ± 0.02
MUC6 $\leftrightarrow$ PROM1	1.09 ± 0.02	1.13 ± 0.09	1.19 ± 0.04
**SST** $\leftrightarrow$ MUC6	1.000 ± 0.005	1.00 ± 0.01	0.99 ± 0.01
**SST** $\leftrightarrow$ PROM1	0.95 ± 0.02	0.99 ± 0.01	1.00 ± 0.03
**SST** $\leftrightarrow$ EPCAM	1.00 ± 0.02	1.00 ± 0.01	1.02 ± 0.01
**GLUC** $\leftrightarrow$ MUC6	0.996 ± 0.002	1.000 ± 0.008	1.000 ± 0.003
**GLUC** $\leftrightarrow$ PROM1	1.005 ± 0.003	0.990 ± 0.007	1.000 ± 0.005
**GLUC** $\leftrightarrow$ EPCAM	0.98 ± 0.01	1.000 ± 0.004	1.000 ± 0.004
**INS** $\leftrightarrow$ MUC6	1.00 ± 0.04	1.00 ± 0.03	0.94 ± 0.03
**INS** $\leftrightarrow$ PROM1	1.00 ± 0.08	0.91 ± 0.05	1.12 ± 0.06
**INS** $\leftrightarrow$ EPCAM	1.2 ± 0.2	1.00 ± 0.04	1.17 ± 0.07
ARX $\leftrightarrow$ MUC6	1.00 ± 0.03	1.07 ± 0.03	1.00 ± 0.04
ARX $\leftrightarrow$ PROM1	1.2 ± 0.1	1.00 ± 0.04	1.1 ± 0.1
ARX $\leftrightarrow$ EPCAM	1.4 ± 0.3	1.00 ± 0.04	1.2 ± 0.1
ARX $\leftrightarrow$ **SST**	1.00 ± 0.02	1.000 ± 0.005	1.00 ± 0.01
ARX $\leftrightarrow$ **GLUC**	1.000 ± 0.004	1.000 ± 0.004	1.01 ± 0.01
ARX $\leftrightarrow$ **INS**	1.0 ± 0.2	1.00 ± 0.04	0.89 ± 0.05

Supplementary Table 7: cDNA primers. Here m = 2-O-Me base, + = LNA base.

Gene	Reference number	Probe name	Probe sequence (5' –>3')
VEGFB	NM_001243733.1	VEGFB_1	cmugmucmugmgcmutmcam cagcactgtcct
VEGFB	NM_001243733.1	VEGFB_2	gccagctgcaggagtgcggcgagca
VEGFB	NM_001243733.1	VEGFB_3	cagctgtttggccacggtgcccatg
VEGFB	NM_001243733.1	VEGFB_4	gtgctgcccagtgggcacacactcc
VEGFB	NM_001243733.1	VEGFB_5	tcaccttcgcagcttcggcacctg
VEGFC	NM_005429.4	VEGFC_1	gmgcmctmugmagmagmagma ggcactgtaat
VEGFC	NM_005429.4	VEGFC_2	gactcgaaggcggcggcggcggcgg
VEGFC	NM_005429.4	VEGFC_3	ctgttatgttgccagcctcctttcc
VEGFC	NM_005429.4	VEGFC_4	tctgttgagtcatctccagcatccg
VEGFC	NM_005429.4	VEGFC_5	gccttctggcggttcgtacatggcc
MUC1	NM_001018016.2	MUC1_1	tmugmugmuamagmagmagmg ctgctgccacc
MUC1	NM_001018016.2	MUC1_2	tgtaagcactgtgaggagcagcagc
MUC1	NM_001018016.2	MUC1_3	agtagtcggtgctgggatcttccag
MUC1	NM_001018016.2	MUC1_4	ttttatactgattgaactgtgtctc
MUC1	NM_001018016.2	MUC1_5	actgacagacagccaaggcaatgag
MUC6	NM_005961.2	MUC6_1	gmgtmggmutmggmgcmctmgt ggtgcttg
MUC6	NM_005961.2	MUC6_2	gcactggcgggagtactccgacagg
MUC6	NM_005961.2	MUC6_3	tggcagccacttggcttctccgttg
MUC6	NM_005961.2	MUC6_4	ggtgggcacgcaggcaacaccggtg
MUC6	NM_005961.2	MUC6_5	ggagcagttgtagcagccttcgatg
MUC6	NM_005961.2	MUC6_6	ggcttggctggtcccgctggtggtc
MUC6	NM_005961.2	MUC6_7	gtagctgtgctgaatgagctgtggg
MUC13	NM_033049.3	MUC13_1	tmcamgcmacmgcmatmutmca gatcttggtg
MUC13	NM_033049.3	MUC13_2	tggtatcagctgcagctactgtagg
MUC13	NM_033049.3	MUC13_3	ggtctccaataaagcggtgccagtg
MUC13	NM_033049.3	MUC13_4	atcgcatgctaaaccattgaggcag
MUC13	NM_033049.3	MUC13_5	aatagtgaggatcagctgaaatttg
MUC13	NM_033049.3	MUC13_6	gctgtgtcttgaatagggattttgc
MUC15	NM_001135091.1	MUC15_1	cmatmcamuamagmgtmucmc ggtgcattgtc
MUC15	NM_001135091.1	MUC15_2	ttttaaaaacttctgcaatgttctg
MUC15	NM_001135091.1	MUC15_3	ttagactgcccaaagaatgctctgc
MUC15	NM_001135091.1	MUC15_4	gttttcacggtgtcattgaccaaag
MUC15	NM_001135091.1	MUC15_5	gaattattggtgaattttaaggtag

MUC16	NM_024690.2	MUC16_1	amutmccmcamgtmuamagmg gctcatttctg
MUC16	NM_024690.2	MUC16_2	catccatggactgagatggagatac
MUC16	NM_024690.2	MUC16_3	ggaaccattggagatgtggctgtag
MUC16	NM_024690.2	MUC16_4	catggtgacagatcttgtggaggtg
MUC16	NM_024690.2	MUC16_5	gagggagtggttgatgtgtctaatg
MUC16	NM_024690.2	MUC16_6	aatccagaagtcagggaggaagttg
MUC16	NM_024690.2	MUC16_7	aggtagacagcctggtgacaacttc
MUC16	NM_024690.2	MUC16_8	tgacttcagaggcagccagtatttc
MUC16	NM_024690.2	MUC16_9	actagaactagtgaccagagaggtc
MUC16	NM_024690.2	MUC16_10	cagaactagtgatctgtgaagtcac
MUC16	NM_024690.2	MUC16_11	ccagctctttgatgccattggtcag
MUC16	NM_024690.2	MUC16_12	aggtcagtctgcagccagagtacag
MUC16	NM_024690.2	MUC16_13	ccatggctgttgtggcttctgacag
MUC20	NM_152673.3	MUC20_1	gmgamagmugmagmgcmgcmg tgggcgtggag
MUC20	NM_152673.3	MUC20_2	tttccagagcggcgtggcccggtgc
MUC20	NM_152673.3	MUC20_3	tgtgagtgtctttgcctcttcagag
MUC20	NM_152673.3	MUC20_4	ttgcttcagtggagtcaggcagagc
MUC20	NM_152673.3	MUC20_5	cagcaaaggaagttgttttgcccac
SOD1	NM_000454.4	SOD1_1	cmccmcamcamccmutmcamct ggtccattac
SOD1	NM_000454.4	SOD1_2	atagaggattaaagtgaggacctgc
SOD1	NM_000454.4	SOD1_3	ctgagagtgagatcacagaatcttc
SOD1	NM_000454.4	SOD1_4	caccacaagccaaacgacttccagc
MUTYH	NM_001048171.1	MUTYH_1	tmacmagmgtmccmctmggmct gttggccctg
MUTYH	NM_001048171.1	MUTYH_2	tggcctgactgttgttcttagcatg
MUTYH	NM_001048171.1	MUTYH_3	tcatcttctgcccgtcttctccatg
MUTYH	NM_001048171.1	MUTYH_4	aatagtagcccaggccagcccagag
MUTYH	NM_001048171.1	MUTYH_5	gctgggaaacaagggtgctgctggg
MUTYH	NM_001048171.1	MUTYH_6	ggtggcactgtccagtgttgggagc
OGG1	NM_002542.5	OGG1_1	cmacmcamctmccmagmugmu gcaggactttg
OGG1	NM_002542.5	OGG1_2	aacatctagctggaagtacttgcgc
OGG1	NM_002542.5	OGG1_3	tggcacagccgctccaccatgccag
OGG1	NM_002542.5	OGG1_4	aggcaggatgcagagggccttgtgg
OGG1	NM_002542.5	OGG1_5	ccggaaaaagtttcccagttccttg
TP53	NM_000546.5	TP53_1	tmutmatmggmcgmggmagmg tagactgaccc
TP53	NM_000546.5	TP53_2	aacgttgttttcaggaagtagtttc
TP53	NM_000546.5	TP53_3	gtgtggaatcaacccacagctgcac
TP53	NM_000546.5	TP53_4	agtgtttctgtcatccaaatactcc

TP53	NM_000546.5	TP53_5	cctcaaagctgttccgtcccagtag
TP53	NM_000546.5	TP53_6	gcccacggatctgaagggtgaaata
CDKN2A	NM_000077.4	CDKN2A_1	amggmutmucmucmagmagmc ctctctggttc
CDKN2A	NM_000077.4	CDKN2A_2	ccgggccgcggccgtggccagcca
CDKN2A	NM_000077.4	CDKN2A_3	gctgcccatcatcatgacctggatc
CDKN2A	NM_000077.4	CDKN2A_4	gcaccaccagcgtgtccaggaagcc
CDKN2A	NM_000077.4	CDKN2A_5	aggtaccgtgcgacatcgcgatggc
CDKN1A	NM_000389.4	CDKN1A_1	gmgamgamagmatmcamgcmc ggcgtttggag
CDKN1A	NM_000389.4	CDKN1A_2	gcgcatcacagtcgcggctcagctg
CDKN1A	NM_000389.4	CDKN1A_3	tagagcttgggcaggccaaggcccc
CDKN1A	NM_000389.4	CDKN1A_4	tgagcgaggcacaagggtacaagac
TOP2A	NM_001067.3	TOP2A_1	cmatmctmutmugmgtmggma aataacaatcg
TOP2A	NM_001067.3	TOP2A_2	ataccttttccattattccatatac
TOP2A	NM_001067.3	TOP2A_3	ttactggcagtttatttccattaag
TOP2A	NM_001067.3	TOP2A_4	gtttaactggacttgggccttaaac
TOP2A	NM_001067.3	TOP2A_5	tttagcttcctttgatgtgctggtg
TOP2A	NM_001067.3	TOP2A_6	gggcaacctttacttctcgcttgtc
TOP2A	NM_001067.3	TOP2A_7	gttcaaaaaagtctcttagaatatc
TOP2A	NM_001067.3	TOP2A_8	gtttcttttctaatctttgttttag
MKI67	NM_001145966.1	MKI67_1	amctmctmugmutmutmccmu gatggttgagg
MKI67	NM_001145966.1	MKI67_2	aacgatcaataatagttattacatc
MKI67	NM_001145966.1	MKI67_3	gtgtttgttttgcaccaagttttac
MKI67	NM_001145966.1	MKI67_4	ccttatatgtctcaaaaggtctttc
MKI67	NM_001145966.1	MKI67_5	gagttcccataaatgctttaatgtc
MKI67	NM_001145966.1	MKI67_6	tgagccgtcgcttggagcttgctgg
MKI67	NM_001145966.1	MKI67_7	ctagtgattgggcctcttccttagg
MKI67	NM_001145966.1	MKI67_8	ctgctggttttggtgtgtccatggc
MKI67	NM_001145966.1	MKI67_9	ttccactgtcaggtgtttgctttgg
MKI67	NM_001145966.1	MKI67_10	tggagcgcagggatattcccttatt
EPCAM	NM_002354.2	EPCAM_1	T+TA+CG+GC+CA+GC +TTGTAGTTTTCACA
EPCAM	NM_002354.2	EPCAM_2	ccaagttttgagccattcatttctg
EPCAM	NM_002354.2	EPCAM_3	gttctcactcgctcagagcaggtta
EPCAM	NM_002354.2	EPCAM_4	agaattttgaaccagatcaatagtg
EPCAM	NM_002354.2	EPCAM_5	tcaggtgctttttcatcaacataat
EPCAM	NM_002354.2	EPCAM_6	ttatatagttatgcattgagttccc
PROM1	NM_001145848.1	PROM1_1	T+GA+TT+TG+CC +ACAAAACCATAGAAGA
PROM1	NM_001145848.1	PROM1_2	atttgttgcaggcaattcataattc

PROM1	NM_001145848.1	PROM1_3	aatccctgcttcatagtagacaatc
PROM1	NM_001145848.1	PROM1_4	gcctctttggtctccttgatcgctg
PROM1	NM_001145848.1	PROM1_5	gttgctattcagctggcttagagac
PROM1	NM_001145848.1	PROM1_6	tggaattcaagacccttttgatacc
PROM1	NM_001145848.1	PROM1_7	taatccaactccaaccatgaggaag
PROM1	NM_001145848.1	PROM1_8	atttattaaatagcttcccagagag
PROM1	NM_001145848.1	PROM1_9	cagaaagatattaagatttaccttc
PROM1	NM_001145848.1	PROM1_10	tgtgatgaagttctgagcaaaatcc
PROM1	NM_001145848.1	PROM1_11	gtcgataatgtagctacacagaaag
PROM1	NM_001145848.1	PROM1_12	gatctttatgataaccattattacc
SST	NM_001048.3	SST_1	C+AG+AC+AG+CA+GC +TC+TGCCAAGAAGTA
SST	NM_001048.3	SST_2	acagcccagggccaggacgatggac
SST	NM_001048.3	SST_3	cctgctcagcagcctgggacagatc
SST	NM_001048.3	SST_4	ctaacaggatgtgaaagtcttccag
ARX	NM_139058.2	ARX_1	GG+CCGCG+GT+CG +ACGCGCTGGTCAGG
ARX	NM_139058.2	ARX_2	cagtaggaggagagcaaagttggag
ARX	NM_139058.2	ARX_3	caggcgccgcagcttgggcggcagg
ARX	NM_139058.2	ARX_4	gctcacctgcggcgcctggctgatc
ARX	NM_139058.2	ARX_5	cgtcgtcctccagcagttcctcttc
ARX	NM_139058.2	ARX_6	cgccgtccttgccctcagcgtcttc
ARX	NM_139058.2	ARX_7	cttctcccgcttgcgccacttggcc
ARX	NM_139058.2	ARX_8	cggaggcggaggtaggctcgggaag
ARX	NM_139058.2	ARX_9	ttagcacacctccttgcccgtgctg
PDX1	NM_000209.3	PDX1_1	T+GT+TC+TC+CT+CC +GGCTCCGCAGCGTA
PDX1	NM_000209.3	PDX1_2	gctgccctgctccagcgcgcccagg
PDX1	NM_000209.3	PDX1_3	ttttccacttcatgcggcggttttg
PDX1	NM_000209.3	PDX1_4	gcggcggcggcggcagcgccagaag
PDX1	NM_000209.3	PDX1_5	gtggttcctgcggccgccgaggcgc
NEUROD1	NM_002500.4	NEUROD1_1	amutmutmagmggmagmacmg gaaagacttta
NEUROD1	NM_002500.4	NEUROD1_2	gcctcgtgctcctcgtcctgagaac
NEUROD1	NM_002500.4	NEUROD1_3	tccgtgcatgcggttccgctcccgg
NEUROD1	NM_002500.4	NEUROD1_4	taagcccttgcaaagcgtctgaacg
NEUROD1	NM_002500.4	NEUROD1_5	gaagggctccagcgctgcgctgtag
NEUROD1	NM_002500.4	NEUROD1_6	tgatccgtggctttgggcccctgcc
NEUROG3	NM_020999.3	NEUROG3_1	gmggmugmgtmggmcamgaga ggaggcctaa
NEUROG3	NM_020999.3	NEUROG3_2	ccgctccgtctcacgggtcacttgg
NEUROG3	NM_020999.3	NEUROG3_3	cattcgattgcgctcgcggtcgttg
NEUROG3	NM_020999.3	NEUROG3_4	cgcagtgcggcgccggcggctccag

NEUROG3	NM_020999.3	NEUROG3_5	cctttcacagaaaatctgagaaagc
INS	NM_000207.2	INS	G+CA+CC+AG+GGC +CCC+CGCCCAGCTCCA
GCG	NM_002054.4	GCG	G+TC+TC+TC+AA+AT +TC+ATCGTGACGTTT

Supplementary Table 8: Padlock Probes.

Gene	Reference number	Probe name	Probe sequence (5' –>3')
VEGFB	NM_001243733.1	VEGFB_1	acctaaaaaaaaaggacaT GTGTCTATTTAGTGGAT CCCCTAGATGTAACGCT ATCGTCTTGTGCTGTAT GATCGTCCagccagtgt gaatgcag
VEGFB	NM_001243733.1	VEGFB_2	gccgcctgctgctcgccT GTGTCTATTTAGTGGAT CCCCTAGATGTAACGCT ATCGTCTTGTGCTGTAT GATCGTCCatgagccct ctgctcc
VEGFB	NM_001243733.1	VEGFB_3	gactgtggagctcatggT GTGTCTATTTAGTGGAT CCCCTAGATGTAACGCT ATCGTCTTGTGCTGTAT GATCGTCCgaggtggtg gtgcccctt
VEGFB	NM_001243733.1	VEGFB_4	ctgacgatggcctggagT GTGTCTATTTAGTGGAT CCCCTAGATGTAACGCT ATCGTCTTGTGCTGTAT GATCGTCCctgtggtgg ctgctgcc
VEGFB	NM_001243733.1	VEGFB_5	gacacctgcaggtgccgT GTGTCTATTTAGTGGAT CCCCTAGATGTAACGCT ATCGTCTTGTGCTGTAT GATCGTCCgcttagagc tcaaccca
VEGFC	NM_005429.4	VEGFC_1	ttatttgaaattacagtC CTCAATGCTGCTGCTGT ACCCTAGATGTAACGCT ATCGTCCTAGATGTTCC GCTATTGTgctacctca gcaagacg
VEGFC	NM_005429.4	VEGFC_2	ctcgcgaggcgcccgccC CTCAATGCTGCTGCTGT ACCCTAGATGTAACGCT ATCGTCCTAGATGTTCC GCTATTGTtgcgctgct cccgggtc

VEGFC	NM_005429.4	VEGFC_3	agtgtcagctaaggaaaC CTCAATGCTGCTGCTGT ACCCTAGATGTAACGCT ATCGTCCTAGATGTTCC GCTATTGTatattggaa aatgtaca
VEGFC	NM_005429.4	VEGFC_4	ttatgttttcctcggatC CTCAATGCTGCTGCTGT ACCCTAGATGTAACGCT ATCGTCCTAGATGTTCC GCTATTGTcctggctca ggaagatt
VEGFC	NM_005429.4	VEGFC_5	gctgttacagacggccaC CTCAATGCTGCTGCTGT ACCCTAGATGTAACGCT ATCGTCCTAGATGTTCC GCTATTGTccaccacca aacatgca
MUC1	NM_001018016.2	MUC1_1	gtttctgcaggtaatggA GTCGGAAGTACTACTCT CTTCTACGATTTTACCA GTTGCCCTAGATGTTCC GCTATTGTgtagcccct atgagaag
MUC1	NM_001018016.2	MUC1_2	ctttcttcctgctgctgA GTCGGAAGTACTACTCT CTTCTACGATTTTACCA GTTGCCCTAGATGTTCC GCTATTGTaccgggcac ccagtctc
MUC1	NM_001018016.2	MUC1_3	tttaattcctctctggaA GTCGGAAGTACTACTCT CTTCTACGATTTTACCA GTTGCCCTAGATGTTCC GCTATTGTctactgaga agaatgct
MUC1	NM_001018016.2	MUC1_4	gtccacgacgtggagacA GTCGGAAGTACTACTCT CTTCTACGATTTTACCA GTTGCCCTAGATGTTCC GCTATTGTgagaaggta ccatcaat

MUC1	NM_001018016.2	MUC1_5	gccattgtctatctcatA GTCGGAAGTACTACTCT CTTCTACGATTTTACCA GTTGCCCTAGATGTTCC GCTATTGTgtgttctgg ttgcgctg
MUC6	NM_005961.2	MUC6_1	ccaaccccagcaagcacA GTACGCATGAATCCGTA GTAGTAGCCGTGACTAT CGTCTGCTCCACTGTTA CTAGATTGagacgacag gcccacgt
MUC6	NM_005961.2	MUC6_2	agttgtgccaccctgtcA GTACGCATGAATCCGTA GTAGTAGCCGTGACTAT CGTCTGCTCCACTGTTA CTAGATTGcaggcccac agaacagc
MUC6	NM_005961.2	MUC6_3	gtggtcaccaacaacggA GTACGCATGAATCCGTA GTAGTAGCCGTGACTAT CGTCTGCTCCACTGTTA CTAGATTGtgatctctc aggacgag
MUC6	NM_005961.2	MUC6_4	cagatgctggccaccggA GTACGCATGAATCCGTA GTAGTAGCCGTGACTAT CGTCTGCTCCACTGTTA CTAGATTGcctgtgccc ccacatgc
MUC6	NM_005961.2	MUC6_5	ccaggcagcaacatcgaA GTACGCATGAATCCGTA GTAGTAGCCGTGACTAT CGTCTGCTCCACTGTTA CTAGATTGgccagccac agagcgtc
MUC6	NM_005961.2	MUC6_6	acaatgacgctgaccacA GTACGCATGAATCCGTA GTAGTAGCCGTGACTAT CGTCTGCTCCACTGTTA CTAGATTGccacccaca cagcgcca

MUC6	NM_005961.2	MUC6_7	gggaccagccaaacccaA GTACGCATGAATCCGTA GTAGTAGCCGTGACTAT CGTCTGCTCCACTGTTA CTAGATTGtgacagtga ccaccagt
MUC13	NM_033049.3	MUC13_1	acatctctgtcaccaagA GTCGGAAGTACTACTCT CTCCTAGATGTAACGCT ATCGTTCTGTCTGTTGA GTTAGTCCtaattctta ctgtaagc
MUC13	NM_033049.3	MUC13_2	gcgactagtggtcctacA GTCGGAAGTACTACTCT CTCCTAGATGTAACGCT ATCGTTCTGTCTGTTGA GTTAGTCCtaacaacca cagaaact
MUC13	NM_033049.3	MUC13_3	tcagggcctcccactggA GTCGGAAGTACTACTCT CTCCTAGATGTAACGCT ATCGTTCTGTCTGTTGA GTTAGTCCcagaagaca atcaatca
MUC13	NM_033049.3	MUC13_4	actgcggatgactgcctA GTCGGAAGTACTACTCT CTCCTAGATGTAACGCT ATCGTTCTGTCTGTTGA GTTAGTCCattatggct gtaaccag
MUC13	NM_033049.3	MUC13_5	gactgtaaggacaaattA GTCGGAAGTACTACTCT CTCCTAGATGTAACGCT ATCGTTCTGTCTGTTGA GTTAGTCCttggctaca gtggactc
MUC13	NM_033049.3	MUC13_6	gacagccagatgcaaaaA GTCGGAAGTACTACTCT CTCCTAGATGTAACGCT ATCGTTCTGTCTGTTGA GTTAGTCCggataacgg cctccaga

MUC15	NM_001135091.1	MUC15_1	tctgcgattagacaatgA GTACGCATGAATCCGTA GTTCTACGATTTTACCA GTTGCCCTAGATGTTCC GCTATTGTgacagaaat gaaccagt
MUC15	NM_001135091.1	MUC15_2	ataaacacaacacagaaA GTACGCATGAATCCGTA GTTCTACGATTTTACCA GTTGCCCTAGATGTTCC GCTATTGTgaaaagaaa atcaagac
MUC15	NM_001135091.1	MUC15_3	agtaactcatcagcagaA GTACGCATGAATCCGTA GTTCTACGATTTTACCA GTTGCCCTAGATGTTCC GCTATTGTgaataacag atttctcc
MUC15	NM_001135091.1	MUC15_4	ttcacttggtctttggtA GTACGCATGAATCCGTA GTTCTACGATTTTACCA GTTGCCCTAGATGTTCC GCTATTGTctctgtctt cagaaaac
MUC15	NM_001135091.1	MUC15_5	actctacagcctaccttA GTACGCATGAATCCGTA GTTCTACGATTTTACCA GTTGCCCTAGATGTTCC GCTATTGTcttatcaag aaaaaaca
MUC16	NM_024690.2	MUC16_1	ggtattctcccaacagaC CTCAATGCTGCTGCTGT ACCGTGCGCCTGGTAGC AATTACCTAGATGTTCC GCTATTGTcagtgtcct tgtggatg
MUC16	NM_024690.2	MUC16_2	tcaatgtcagtatctccC CTCAATGCTGCTGCTGT ACCGTGCGCCTGGTAGC AATTACCTAGATGTTCC GCTATTGTtggccaaag aaagtgtc

MUC16	NM_024690.2	MUC16_3	tcagaaccatctacagcC CTCAATGCTGCTGCTGT ACCGTGCGCCTGGTAGC AATTACCTAGATGTTCC GCTATTGTccactctcc cagtcagc
MUC16	NM_024690.2	MUC16_4	atgtcaccggacacctcC CTCAATGCTGCTGCTGT ACCGTGCGCCTGGTAGC AATTACCTAGATGTTCC GCTATTGTgcactgaaa agcccaca
MUC16	NM_024690.2	MUC16_5	agtacctttacattagaC CTCAATGCTGCTGCTGT ACCGTGCGCCTGGTAGC AATTACCTAGATGTTCC GCTATTGTctgggtcta catcagag
MUC16	NM_024690.2	MUC16_6	atgacacagccaacttcC CTCAATGCTGCTGCTGT ACCGTGCGCCTGGTAGC AATTACCTAGATGTTCC GCTATTGTccttcccag aaactatg
MUC16	NM_024690.2	MUC16_7	gacatctccgatgaagtC CTCAATGCTGCTGCTGT ACCGTGCGCCTGGTAGC AATTACCTAGATGTTCC GCTATTGTagtccacaa tgtcacta
MUC16	NM_024690.2	MUC16_8	acctcagttgaaatactC CTCAATGCTGCTGCTGT ACCGTGCGCCTGGTAGC AATTACCTAGATGTTCC GCTATTGTctccaaatc tgagtggc
MUC16	NM_024690.2	MUC16_9	ccaggagtggtgacctcC CTCAATGCTGCTGCTGT ACCGTGCGCCTGGTAGC AATTACCTAGATGTTCC GCTATTGTctgtttcac ctgaggta

MUC16	NM_024690.2	MUC16_10	gcaccagagatggtgacC CTCAATGCTGCTGCTGT ACCGTGCGCCTGGTAGC AATTACCTAGATGTTCC GCTATTGTcaacaatct cacctggt
MUC16	NM_024690.2	MUC16_11	gagctgagccaactgacC CTCAATGCTGCTGCTGT ACCGTGCGCCTGGTAGC AATTACCTAGATGTTCC GCTATTGTgagagcggc tgtactgg
MUC16	NM_024690.2	MUC16_12	agtgtcggccttctgtaC CTCAATGCTGCTGCTGT ACCGTGCGCCTGGTAGC AATTACCTAGATGTTCC GCTATTGTccatgttca agaacacc
MUC16	NM_024690.2	MUC16_13	ttcctgcctcctctgtcC CTCAATGCTGCTGCTGT ACCGTGCGCCTGGTAGC AATTACCTAGATGTTCC GCTATTGTccaagccag ccaccaca
MUC20	NM_152673.3	MUC20_1	ctccaccgggaactccaA GTACGCATGAATCCGTA GTAGTAGCCGTGACTAT CGTCTCCTAGATGTTCC GCTATTGTaaaggctga tgcagcag
MUC20	NM_152673.3	MUC20_2	gctatgactctagcaccA GTACGCATGAATCCGTA GTAGTAGCCGTGACTAT CGTCTCCTAGATGTTCC GCTATTGTacgacacag aagtgccc
MUC20	NM_152673.3	MUC20_3	accgatgacagctctgaA GTACGCATGAATCCGTA GTAGTAGCCGTGACTAT CGTCTCCTAGATGTTCC GCTATTGTtctttgaca ccctttgc

MUC20	NM_152673.3	MUC20_4	tccgatccaccagctctA GTACGCATGAATCCGTA GTAGTAGCCGTGACTAT CGTCTCCTAGATGTTCC GCTATTGTtgaaggcct cgtccacc
MUC20	NM_152673.3	MUC20_5	gctgggtcagcagtgggA GTACGCATGAATCCGTA GTAGTAGCCGTGACTAT CGTCTCCTAGATGTTCC GCTATTGTctccggtct ccatagag
SOD1	NM_000454.4	SOD1_1	agcagaaggaaagtaatA GTCGGAAGTACTACTCT CTAGTAGCCGTGACTAT CGTCTCCTAGATGTTCC GCTATTGTgggcatcat caatttcg
SOD1	NM_000454.4	SOD1_2	ctgtaccagtgcaggtcA GTCGGAAGTACTACTCT CTAGTAGCCGTGACTAT CGTCTCCTAGATGTTCC GCTATTGTggagataat acagcagg
SOD1	NM_000454.4	SOD1_3	tgtgtctattgaagattA GTCGGAAGTACTACTCT CTAGTAGCCGTGACTAT CGTCTCCTAGATGTTCC GCTATTGTaaagatggt gtggccga
SOD1	NM_000454.4	SOD1_4	gacaggaaacgctggaaA GTCGGAAGTACTACTCT CTAGTAGCCGTGACTAT CGTCTCCTAGATGTTCC GCTATTGTaatgaagaa agtacaaa
MUTYH	NM_001048171.1	MUTYH_1	gttttccgtgtgtatcaC CTCAATGCTGCTGCTGT ACCGTGCGCCTGGTAGC AATTATCTGTCTGTTGA GTTAGTCCccaccgcca tgaaaaag

MUTYH	NM_001048171.1	MUTYH_2	gggaggcagaagcatgcC CTCAATGCTGCTGCTGT ACCGTGCGCCTGGTAGC AATTATCTGTCTGTTGA GTTAGTCCaggcagcca gccaggaa
MUTYH	NM_001048171.1	MUTYH_3	cgggacctaccatggagC CTCAATGCTGCTGCTGT ACCGTGCGCCTGGTAGC AATTATCTGTCTGTTGA GTTAGTCCggtacgacc aagagaaa
MUTYH	NM_001048171.1	MUTYH_4	gaggtgaatcaactctgC CTCAATGCTGCTGCTGT ACCGTGCGCCTGGTAGC AATTATCTGTCTGTTGA GTTAGTCCccagtgctt ccctggag
MUTYH	NM_001048171.1	MUTYH_5	attggtgctgatcccagC CTCAATGCTGCTGCTGT ACCGTGCGCCTGGTAGC AATTATCTGTCTGTTGA GTTAGTCCtgtgccgtg tccgagcc
MUTYH	NM_001048171.1	MUTYH_6	gtggaggagtgtgctcccC CTCAATGCTGCTGCTGT ACCGTGCGCCTGGTAGC AATTATCTGTCTGTTGA GTTAGTCCtgtcgggca gtcctgac
OGG1	NM_002542.5	OGG1_1	tccggtggagggagcaaT GTGTCTATTTAGTGGAT CCTCTACGATTTTACCA GTTGCGCTCCACTGTTA CTAGATTGgccttctgg acaatctt
OGG1	NM_002542.5	OGG1_2	gctggaggccgtgcgcaT GTGTCTATTTAGTGGAT CCTCTACGATTTTACCA GTTGCGCTCCACTGTTA CTAGATTGaggcccaca ccagacga

OGG1	NM_002542.5	OGG1_3	cgcccgcatcactggcaT GTGTCTATTTAGTGGAT CCTCTACGATTTTACCA GTTGCGCTCCACTGTTA CTAGATTGtcctccaac aacaacat
OGG1	NM_002542.5	OGG1_4	atatgaggaggcccacaT GTGTCTATTTAGTGGAT CCTCTACGATTTTACCA GTTGCGCTCCACTGTTA CTAGATTGcagctacga gagtcctc
OGG1	NM_002542.5	OGG1_5	cccagaccaacaaggaaT GTGTCTATTTAGTGGAT CCTCTACGATTTTACCA GTTGCGCTCCACTGTTA CTAGATTGggcgaaggg accgagcc
TP53	NM_000546.5	TP53_1	cacctgaagtccaaaaaT GTGTCTATTTAGTGGAT CCCCTAGATGTAACGCT ATCGTGCTCCACTGTTA CTAGATTGgcagggctc actccagc
TP53	NM_000546.5	TP53_2	ttcagacctatggaaacT GTGTCTATTTAGTGGAT CCCCTAGATGTAACGCT ATCGTGCTCCACTGTTA CTAGATTGctgagtcag gaaacatt
TP53	NM_000546.5	TP53_3	gacctgccctgtgcagcT GTGTCTATTTAGTGGAT CCCCTAGATGTAACGCT ATCGTGCTCCACTGTTA CTAGATTGttttgccaa ctggccaa
TP53	NM_000546.5	TP53_4	aaatttgcgtgtggagtT GTGTCTATTTAGTGGAT CCCCTAGATGTAACGCT ATCGTGCTCCACTGTTA CTAGATTGcttatccga gtggaagg

TP53	NM_000546.5	TP53_5	ctccagtggtaatctacT GTGTCTATTTAGTGGAT CCCCTAGATGTAACGCT ATCGTGCTCCACTGTTA CTAGATTGatcatcaca ctggaaga
TP53	NM_000546.5	TP53_6	ggatggagaatatttcaT GTGTCTATTTAGTGGAT CCCCTAGATGTAACGCT ATCGTGCTCCACTGTTA CTAGATTGccaaagaag aaaccact
CDKN2A	NM_000077.4	CDKN2A_1	acatccccgattgaaagA GTCGGAAGTACTACTCT CTAGTAGCCGTGACTAT CGTCTGCTCCACTGTTA CTAGATTGcgcggaagg tccctcag
CDKN2A	NM_000077.4	CDKN2A_2	cggctgactggctggccA GTCGGAAGTACTACTCT CTAGTAGCCGTGACTAT CGTCTGCTCCACTGTTA CTAGATTGagcagcatg gagcctt
CDKN2A	NM_000077.4	CDKN2A_3	ggtcggaggccgatccaA GTCGGAAGTACTACTCT CTAGTAGCCGTGACTAT CGTCTGCTCCACTGTTA CTAGATTGacgcaccga atagttac
CDKN2A	NM_000077.4	CDKN2A_4	gctgcccgggagggcttA GTCGGAAGTACTACTCT CTAGTAGCCGTGACTAT CGTCTGCTCCACTGTTA CTAGATTGcccgacccg tgcacgac
CDKN2A	NM_000077.4	CDKN2A_5	ctgaggagctgggccatA GTCGGAAGTACTACTCT CTAGTAGCCGTGACTAT CGTCTGCTCCACTGTTA CTAGATTGtctgcccgt ggacctgg

CDKN1A	NM_000389.4	CDKN1A_1	gacagatttctaccactCCTCAATGCTGCTGCTGTACAGTAGCCGTGACTATCGTCTCCTAGATGTTCCGCTATTGTcggcggcagaccagcat
CDKN1A	NM_000389.4	CDKN1A_2	tggacagcgagcagctgCCTCAATGCTGCTGCTGTACAGTAGCCGTGACTATCGTCTCCTAGATGTTCCGCTATTGTccgcctcttcggcccag
CDKN1A	NM_000389.4	CDKN1A_3	gcctgggagcgtgtgcgCCTCAATGCTGCTGCTGTACAGTAGCCGTGACTATCGTCTCCTAGATGTTCCGCTATTGTcactggagggtgacttc
CDKN1A	NM_000389.4	CDKN1A_4	gacctgtcactgtcttgCCTCAATGCTGCTGCTGTACAGTAGCCGTGACTATCGTCTCCTAGATGTTCCGCTATTGTcagaggaagaccatgtg
TOP2A	NM_001067.3	TOP2A_1	agctctttggctcgattAGTCGGAAGTACTACTCTCTCGTGCGCCTGGTAGCAATTAGCTCCACTGTTACTAGATTGacatctttacaatgctc
TOP2A	NM_001067.3	TOP2A_2	aatttaattagtatatgAGTCGGAAGTACTACTCTCTCGTGCGCCTGGTAGCAATTAGCTCCACTGTTACTAGATTGcaattgatccggaaaac
TOP2A	NM_001067.3	TOP2A_3	aaagtctttcttaatggAGTCGGAAGTACTACTCTCTCGTGCGCCTGGTAGCAATTAGCTCCACTGTTACTAGATTGgatccaccaaagatgtc

TOP2A	NM_001067.3	TOP2A_4	tgggtgaagtttaaggcAGTCGGAAGTACTACTCTCTCGTGCGCCTGGTAGCAATTAGCTCCACTGTTACTAGATTGtagaaagcatactaaac
TOP2A	NM_001067.3	TOP2A_5	aaaggtttgggcaccagAGTCGGAAGTACTACTCTCTCGTGCGCCTGGTAGCAATTAGCTCCACTGTTACTAGATTGggaaagtcaaatattac
TOP2A	NM_001067.3	TOP2A_6	ttcaaacggaatgacaaAGTCGGAAGTACTACTCTCTCGTGCGCCTGGTAGCAATTAGCTCCACTGTTACTAGATTGaggttttgtttacttgc
TOP2A	NM_001067.3	TOP2A_7	acggtgttggatattctAGTCGGAAGTACTACTCTCTCGTGCGCCTGGTAGCAATTAGCTCCACTGTTACTAGATTGgtttaaagaaatatgac
TOP2A	NM_001067.3	TOP2A_8	gaactagaaggcctaaaAGTCGGAAGTACTACTCTCTCGTGCGCCTGGTAGCAATTAGCTCCACTGTTACTAGATTGctcaagaagatggtgtg
MKI67	NM_001145966.1	MKI67_1	atcaaggaacagcctcaCCTCAATGCTGCTGCTGTACCGTGCGCCTGGTAGCAATTACTTGTGCTGTATGATCGTCCctgtcctgaagaaaatc
MKI67	NM_001145966.1	MKI67_2	ctaaaacatggagatgtCCTCAATGCTGCTGCTGTACCGTGCGCCTGGTAGCAATTACTTGTGCTGTATGATCGTCCttgatgagcctgtacgg

MKI67	NM_001145966.1	MKI67_3	gcagatgtagtaaaactC CTCAATGCTGCTGCTGT ACCGTGCGCCTGGTAGC AATTACTTGTGCTGTAT GATCGTCCttgttgcaa aatcatgg
MKI67	NM_001145966.1	MKI67_4	atgaaggaaatagaaagC CTCAATGCTGCTGCTGT ACCGTGCGCCTGGTAGC AATTACTTGTGCTGTAT GATCGTCCaaaggagag aaggagag
MKI67	NM_001145966.1	MKI67_5	ggtgatgagaaagacatC CTCAATGCTGCTGCTGT ACCGTGCGCCTGGTAGC AATTACTTGTGCTGTAT GATCGTCCcgcccaaac cagcagga
MKI67	NM_001145966.1	MKI67_6	ccagacaaaaacccagcC CTCAATGCTGCTGCTGT ACCGTGCGCCTGGTAGC AATTACTTGTGCTGTAT GATCGTCCaatcttcac aaccagac
MKI67	NM_001145966.1	MKI67_7	tggccaagaacacctaaC CTCAATGCTGCTGCTGT ACCGTGCGCCTGGTAGC AATTACTTGTGCTGTAT GATCGTCCgaactggga tggagagg
MKI67	NM_001145966.1	MKI67_8	tcagcaggcaaagccatC CTCAATGCTGCTGCTGT ACCGTGCGCCTGGTAGC AATTACTTGTGCTGTAT GATCGTCCtcaggaaac gaacacca
MKI67	NM_001145966.1	MKI67_9	tttacaagcgctccaaaC CTCAATGCTGCTGCTGT ACCGTGCGCCTGGTAGC AATTACTTGTGCTGTAT GATCGTCCatggtgctg ctgatagc

MKI67	NM_001145966.1	MKI67_10	tcggtccctgaaaataaC CTCAATGCTGCTGCTGT ACCGTGCGCCTGGTAGC AATTACTTGTGCTGTAT GATCGTCCaacacaaat tacaagac
EPCAM	NM_002354.2	EPCAM_1	AATGTGTCTGTGAAAACT AGTCGGAAGTACTACTC TCTTCTACGATTTTACC AGTTGCGCTCCACTGTT ACTAGATTGTTGCCGCA GCTCAGGAAG
EPCAM	NM_002354.2	EPCAM_2	gtgatgaaggcagaaatA GTCGGAAGTACTACTCT CTTCTACGATTTTACCA GTTGCGCTCCACTGTTA CTAGATTGtggctgcca aatgtttg
EPCAM	NM_002354.2	EPCAM_3	actgaaataacctgctcA GTCGGAAGTACTACTCT CTTCTACGATTTTACCA GTTGCGCTCCACTGTTA CTAGATTGgaagaacag acaaggac
EPCAM	NM_002354.2	EPCAM_4	gaataatgttatcactaA GTCGGAAGTACTACTCT CTTCTACGATTTTACCA GTTGCGCTCCACTGTTA CTAGATTGacgagtatt ttgtatga
EPCAM	NM_002354.2	EPCAM_5	tttaatttattatgttgA GTCGGAAGTACTACTCT CTTCTACGATTTTACCA GTTGCGCTCCACTGTTA CTAGATTGctggatcct ggtcaaac
EPCAM	NM_002354.2	EPCAM_6	gatgcatagggaactcaA GTCGGAAGTACTACTCT CTTCTACGATTTTACCA GTTGCGCTCCACTGTTA CTAGATTGataaaggag atgggtga

PROM1	NM_001145848.1	PROM1_1	CATTGGCATCTTCTATGG CCTCAATGCTGCTGCTG TACTCTACGATTTTACC AGTTGCGCTCCACTGTT ACTAGATTGGATTTGTA TAATAATAAG
PROM1	NM_001145848.1	PROM1_2	tcctaaggcttggaattC CTCAATGCTGCTGCTGT ACTCTACGATTTTACCA GTTGCGCTCCACTGTTA CTAGATTGccttcatcc acagatgc
PROM1	NM_001145848.1	PROM1_3	tgattatgacaagattgC CTCAATGCTGCTGCTGT ACTCTACGATTTTACCA GTTGCGCTCCACTGTTA CTAGATTGgcatatgaa tccaaaat
PROM1	NM_001145848.1	PROM1_4	gtccatggcaacagcgaC CTCAATGCTGCTGCTGT ACTCTACGATTTTACCA GTTGCGCTCCACTGTTA CTAGATTGgttcttgat gagattaa
PROM1	NM_001145848.1	PROM1_5	cagcatcagattgtctcC CTCAATGCTGCTGCTGT ACTCTACGATTTTACCA GTTGCGCTCCACTGTTA CTAGATTGtcaagtgaa acctgcaa
PROM1	NM_001145848.1	PROM1_6	tgtcgtagcaggtatcaC CTCAATGCTGCTGCTGT ACTCTACGATTTTACCA GTTGCGCTCCACTGTTA CTAGATTGcaacgccaa accacgac
PROM1	NM_001145848.1	PROM1_7	caccggaggcgtcttccC CTCAATGCTGCTGCTGT ACTCTACGATTTTACCA GTTGCGCTCCACTGTTA CTAGATTGcgaggctgt gtctccaa

PROM1	NM_001145848.1	PROM1_8	ctgggaatactatctctC<br>CTCAATGCTGCTGCTGT<br>ACTCTACGATTTTACCA<br>GTTGCGCTCCACTGTTA<br>CTAGATTGtacttacta<br>aatgaaga
PROM1	NM_001145848.1	PROM1_9	attggaaagtctgaaggC<br>CTCAATGCTGCTGCTGT<br>ACTCTACGATTTTACCA<br>GTTGCGCTCCACTGTTA<br>CTAGATTGggaagcata<br>agcagtga
PROM1	NM_001145848.1	PROM1_10	agcttctctggattttgC<br>CTCAATGCTGCTGCTGT<br>ACTCTACGATTTTACCA<br>GTTGCGCTCCACTGTTA<br>CTAGATTGagagtaact<br>aggattct
PROM1	NM_001145848.1	PROM1_11	tgctgttgatgtctttcC<br>CTCAATGCTGCTGCTGT<br>ACTCTACGATTTTACCA<br>GTTGCGCTCCACTGTTA<br>CTAGATTGgccaccgct<br>ctagatac
PROM1	NM_001145848.1	PROM1_12	aaatatggaaaatggtaC<br>CTCAATGCTGCTGCTGT<br>ACTCTACGATTTTACCA<br>GTTGCGCTCCACTGTTA<br>CTAGATTGgaaactata<br>cccatgaa
SST	NM_001048.3	SST_1	GAACTGGCCAAGTACTTC<br>TGTGTCTATTTAGTGGA<br>TCCAGTAGCCGTGACTA<br>TCGTCTCTTGTGCTGTA<br>TGATCGTCCGCTGCCGC<br>GGGGAAGCAG
SST	NM_001048.3	SST_2	gctggctgcgctgtccaT<br>GTGTCTATTTAGTGGAT<br>CCAGTAGCCGTGACTAT<br>CGTCTCTTGTGCTGTAT<br>GATCGTCCtgccgcctc<br>cagtgcgc

SST	NM_001048.3	SST_3	cctggaacctgaagatcTGTGTCTATTTAGTGGATCCAGTAGCCGTGACTATCGTCTCTTGTGCTGTATGATCGTCCcagacggagaatgatgc
SST	NM_001048.3	SST_4	agaatttcttctggaagTGTGTCTATTTAGTGGATCCAGTAGCCGTGACTATCGTCTCTTGTGCTGTATGATCGTCCacgcaaagctggctgca
ARX	NM_139058.2	ARX_1	GCTCTTTTCCACAATGGCTGTGTCTATTTAGTGGATCCCGTGCGCCTGGTAGCAATTACTTGTGCTGTATGATCGTCCCAGCCCGGCATTCGGCAG
ARX	NM_139058.2	ARX_2	caaaagtaaatctccaaTGTGTCTATTTAGTGGATCCCGTGCGCCTGGTAGCAATTACTTGTGCTGTATGATCGTCCtccgagaggcccgagtg
ARX	NM_139058.2	ARX_3	ggccgagctgcacctgcTGTGTCTATTTAGTGGATCCCGTGCGCCTGGTAGCAATTACTTGTGCTGTATGATCGTCCagcagcgccccgttcga
ARX	NM_139058.2	ARX_4	ggacacgctcaagatcaTGTGTCTATTTAGTGGATCCCGTGCGCCTGGTAGCAATTACTTGTGCTGTATGATCGTCCgcggccgccgcggcctg
ARX	NM_139058.2	ARX_5	tgaggacgaggaagaggTGTGTCTATTTAGTGGATCCCGTGCGCCTGGTAGCAATTACTTGTGCTGTATGATCGTCCctggaggacgaagaaga

ARX	NM_139058.2	ARX_6	gctgcacccggaagacgT GTGTCTATTTAGTGGAT CCCGTGCGCCTGGTAGC AATTACTTGTGCTGTAT GATCGTCCcccaaggag gagctgct
ARX	NM_139058.2	ARX_7	ccagaaccgtcgggccaT GTGTCTATTTAGTGGAT CCCGTGCGCCTGGTAGC AATTACTTGTGCTGTAT GATCGTCCcgagtccag gtctggtt
ARX	NM_139058.2	ARX_8	cgccgccgccgccttccT GTGTCTATTTAGTGGAT CCCGTGCGCCTGGTAGC AATTACTTGTGCTGTAT GATCGTCCactgccgct gccgccgc
ARX	NM_139058.2	ARX_9	ccgggcaccagcacgggT GTGTCTATTTAGTGGAT CCCGTGCGCCTGGTAGC AATTACTTGTGCTGTAT GATCGTCCcgcagctca acatcctg
PDX1	NM_000209.3	PDX1_1	GCGGCGCCTACGCTGCGG CCTCAATGCTGCTGCTG TACCGTGCGCCTGGTAG CAATTAGCTCCACTGTT ACTAGATTGGGAAAGGC CAGTGGGCAG
PDX1	NM_000209.3	PDX1_2	cctggcgccctgggcgcC CTCAATGCTGCTGCTGT ACCGTGCGCCTGGTAGC AATTAGCTCCACTGTTA CTAGATTGcgccgccgc acccgttc
PDX1	NM_000209.3	PDX1_3	aagatctggttccaaaaC CTCAATGCTGCTGCTGT ACCGTGCGCCTGGTAGC AATTAGCTCCACTGTTA CTAGATTGtgaccgaga gacacatc

PDX1	NM_000209.3	PDX1_4	tccggcgaggagcttctC CTCAATGCTGCTGCTGT ACCGTGCGCCTGGTAGC AATTAGCTCCACTGTTA CTAGATTGaggactgcg ccgtgacc
PDX1	NM_000209.3	PDX1_5	ccctccagcgtcgcgccC CTCAATGCTGCTGCTGT ACCGTGCGCCTGGTAGC AATTAGCTCCACTGTTA CTAGATTGttagcgcgt cgccacag
NEUROD1	NM_002500.4	NEUROD1_1	AATTCATTTCACTTTAAA AGTACGCATGAATCCGT AGTCGTGCGCCTGGTAG CAATTATCTGTCTGTTG AGTTAGTCCAAATTGCA CAATTTGAGC
NEUROD1	NM_002500.4	NEUROD1_2	cgagtgtctcagttctcA GTACGCATGAATCCGTA GTCGTGCGCCTGGTAGC AATTATCTGTCTGTTGA GTTAGTCCcctccaagc tggacaga
NEUROD1	NM_002500.4	NEUROD1_3	gaaggctaacgcccgggA GTACGCATGAATCCGTA GTCGTGCGCCTGGTAGC AATTATCTGTCTGTTGA GTTAGTCCtttaaattg agacgcat
NEUROD1	NM_002500.4	NEUROD1_4	cctggtctccttcgttcA GTACGCATGAATCCGTA GTCGTGCGCCTGGTAGC AATTATCTGTCTGTTGA GTTAGTCCtcaggcaaa agcccaga
NEUROD1	NM_002500.4	NEUROD1_5	tccgccgcacgcctacaA GTACGCATGAATCCGTA GTCGTGCGCCTGGTAGC AATTATCTGTCTGTTGA GTTAGTCCgtcttccac gttaagcc

NEUROD1	NM_002500.4	NEUROD1_6	tgcagcgacactggcagA GTACGCATGAATCCGTA GTCGTGCGCCTGGTAGC AATTATCTGTCTGTTGA GTTAGTCCtttaccatg cactatcc
NEUROG3	NM_020999.3	NEUROG3_1	TGCGCTCATTTTAGGCCT AGTCGGAAGTACTACTC TCTCCTAGATGTAACGC TATCGTCTTGTGCTGTA TGATCGTCCGAAGTGGG CATTGCAAAG
NEUROG3	NM_020999.3	NEUROG3_2	cgcccactgtccaagtgA GTCGGAAGTACTACTCT CTCCTAGATGTAACGCT ATCGTCTTGTGCTGTAT GATCGTCCgcctcaacc ctcgggtg
NEUROG3	NM_020999.3	NEUROG3_3	cgaaagaaggccaacgaA GTCGGAAGTACTACTCT CTCCTAGATGTAACGCT ATCGTCTTGTGCTGTAT GATCGTCCagcagcgac ggagtcgg
NEUROG3	NM_020999.3	NEUROG3_4	agcttgtacgcgctggaA GTCGGAAGTACTACTCT CTCCTAGATGTAACGCT ATCGTCTTGTGCTGTAT GATCGTCCtgcgcatag cggaccac
NEUROG3	NM_020999.3	NEUROG3_5	ccaggcagtctggctttA GTCGGAAGTACTACTCT CTCCTAGATGTAACGCT ATCGTCTTGTGCTGTAT GATCGTCCtttccgcct gcttgagc
INS	NM_000207.2	INS	AGGTGGGGCAGGTGGAGC CTCAATGCTGCTGCTGT ACTCTACGATTTTACCA GTTGCCCTAGATGTTCC GCTATTGTCCGGGAGGC AGAGGACCTGC

GCG	NM_002054.4	GCG	GAATAACATTGCCAAACG TGTGTCTATTTAGTGGA TCCCGTGCGCCTGGTAG CAATTAGCTCCACTGTT ACTAGATTGGAATACCA AGAGGAACAG

Supplementary Table 9: Detection Probes

Hybridization Cycle	Probe name	Probe sequence (5' –>3')
1	DO_1.1	AGUCGGAAGUACTACTCUCT_FITC
1	DO_1.2	CCUCAATGCUGCTGCTGUAC_Cy3
1	DO_1.3	TGUGTCTATUTAGTGGAUCC_Cy5
1	DO_1.4	AGUACGCAUGAAUCCGUAGT_TR
2	DO_2.1	CGUGCGCCUGGTAGCAAUTA_FITC
2	DO_2.2	AGUAGCCGUGACTATCGUCT_Cy3
2	DO_2.3	TCUACGATUTTACCAGTUGC_Cy5
2	DO_2.4	CCUAGATGUAACGCUAUCGT_TR
3	DO_3.1	CCUAGATGTUCCGCTATUGT_FITC
3	DO_3.2	GCUCCACTGUTACTAGAUTG_Cy3
3	DO_3.3	CTUGTGCTGUATGATCGUCC_Cy5
3	DO_3.4	TCUGTCTGUTGAGUTAGUCC_TR

Supplementary References

[1] Mignardi, M. *et al.* Oligonucleotide gap-fill ligation for mutation detection and sequencing in situ. *Nucleic acids research* **43**, e151–e151 (2015).

[2] Ke, R. *et al.* In situ sequencing for rna analysis in preserved tissue and cells. *Nature methods* **10**, 857 (2013).

[3] Jones, M. C. Simple boundary correction for kernel density estimation. *Statistics and Computing* **3**, 135–146 (1993).

[4] Svensson, V., Teichmann, S. A. & Stegle, O. Spatialde: identification of spatially variable genes. *Nature methods* **15**, 343 (2018).
